# ﻿Morphological and molecular evidence suggests that *Indosasagigantea* and *Acidosasaglauca* (Poaceae, Bambusoideae, Arundinarieae) are conspecific

**DOI:** 10.3897/phytokeys.255.143020

**Published:** 2025-04-24

**Authors:** Zhengyang Niu, Zhuoyu Cai, Jun Yin, Yihua Tong, Nianhe Xia

**Affiliations:** 1 Laboratory of Plant Resources Conservation and Sustainable Utilization & Key Laboratory of National Forestry and Grassland Administration on Plant Conservation and Utilization in Southern China, South China Botanical Garden, Chinese Academy of Sciences, 510650, Guangzhou, China; 2 University of Chinese Academy of Sciences, 100049, Beijing, China; 3 South China National Botanical Garden, Chinese Academy of Sciences, Guangzhou, 510650, China; 4 Co-Innovation Center for Sustainable Forestry in Southern China, Nanjing Forestry University, 210037, Nanjing, China; 5 Bamboo Research Institute, Nanjing Forestry University, 210037, Nanjing, China; 6 Forestry Workstation, Baoxi County, 323724, Longquan, China

**Keywords:** Arundinarieae, morphology, new synonym, plastomes, single-copy orthologous genes, phylogeny

## Abstract

*Indosasagigantea* is a bamboo with great economical value, but its generic designation has been controversial for a long time. This study aims to ascertain whether *I.gigantea* belongs to *Indosasa* or *Acidosasa*, based on morphological and molecular evidence from both plastome and single-copy nuclear orthologous genes. The results of phylogenetic analyses, based on plastid genomes and nuclear gene sequences, both strongly supported that *I.gigantea* is distantly related to other members of *Indosasa*, but clustered with *Acidosasaglauca*, the type species of *Acidosasa*. Further morphological studies demonstrated that *I.gigantea* is conspecific with *A.glauca*. Thus, *I.gigantea* was proposed as a synonym of *A.glauca*. Colour plates as well as a detailed description of this species are also provided.

## ﻿Introduction

*Indosasagigantea* (T. H. Wen) T. H. Wen (1991) is a bamboo native to Fujian Province of China, with great economical value for its tasty bamboo shoots and beautiful figure with erect culms that are 7–17 m tall (Wen 1991; Zheng and Lin 1995; Zhu and Zhao 1996; Liao et al. 2003, 2016; Zhu and Stapleton 2006; Wang 2010). This species has been introduced to neighbouring Zhejiang and Guangdong Provinces (often called 江南笋 jiāng nán sǔn) in recent years according to the historical documents (Wen 1991) and our field investigations. However, the generic designation of *I.gigantea* has been controversial for a long time. Initially, Wen (1983) described this species as a new species of *Sinobambusa* Makino ex Nakai (Nakai 1925), namely *S.gigantea* T. H. Wen, based on vegetative materials. Eight years later, he stated that some newly-collected reproductive materials of this species showed the diagnostic characters for *Indosasa*, i.e. iterauctant or indeterminate inflorescence and six stamens per floret, so he transferred it to *Indosasa* McClure (Wen 1991). However, after re-examining the reproductive materials of *I.gigantea*, Xie and Chen (1993) argued that this species actually had semelauctant and raceme-like inflorescence with true pedicellate spikelets, which matched well with the diagnostic characters of *Acidosasa* B. M. Yang (1981). Thus, they made a new combination for this species as *Acidosasagigantea* (T. H. Wen) Q. Z. Xie & W. Y. Zhang. It seems that Xie and Chen’s opinion was ignored, since many later important floral works and databases adopted *Indosasagigantea* (T. H. Wen) T. H. Wen (Wen et al. 1993; Zhu and Stapleton 2006; Vorontsova et al. 2016; IPNI 2024). However, Zhu and Stapleton (2006) noted that “a different interpretation of the inflorescence can place this species in *Acidosasa*” in their account of *Indosasa* of "Flora of China".

Neither Wen (1991) or Xie and Chen (1993) provided any information of the flowering specimens they examined. As we known, the spikelet type (true spikelet or pseudo-spikelet) is the key character to differentiate *Indosasa* and *Acidosasa*. To ascertain the spikelet type of *I.gigantea*, we visited the herbarium of Zhejiang Forestry Institute (ZJFI) where Wen worked and tried to find the flowering materials of this species. However, despite of an exhaustive search, we could not find any reproductive material of this species, other than the holotype with vegetative organs. Surprisingly to us, the holotype of *I.gigantea* shows a number of characters that are very similar to the type species of *Acidosasa*, i.e. *A.glauca* B. M. Yang, especially the morphology of culm leaf, such as the powdery and setose sheath, arcuate ligule and auricles with well-developed oral setae. To clear the confusions of the taxonomic identity of *Indosasagigantea*, we conducted morphological and phylogenetic studies as below.

## ﻿Materials and methods

### ﻿Morphological study

Voucher specimens were collected during several field trips from 2019–2022 mainly to the type localities of many bamboos including Longquan County of Zhejiang Province (type locality of *Indosasagigantea*) and Jianghua County of Hunan Province (type locality of *Acidosasaglauca*) and were kept in the Herbarium of South China Botanical Garden (IBSC). Some flowering materials (*BH225*, IBSC) were collected at Shaoguan City of Guangdong Province which were named as “江南笋” and we identified them as *Indosasagigantea*. Specimens from the Herbarium of Hunan Normal University (HNNU), IBSC and ZJFI were examined. Herbarium acronyms follow Thiers (2024, updated continuously). Flowering materials were dissected under a stereomicroscope (Mshot-MZ101) and small parts were measured and photographed with the camera attachment (Mshot-MSX2). Terminology follows McClure (1940), Li et al. (2006) and Beentje (2016).

### ﻿Taxon sampling, DNA extraction and sequencing

To ascertain the phylogenetic position of *Indosasagigantea*, phylogenetic analyses, based on sequences of plastomes and single-copy orthologous genes, were conducted. The taxon sampling referred to a previous study of the tribe Arundinarieae by Guo et al. (2021). For plastome-based phylogenetic inference, the ingroup contains two samples of *I.gigantea* (*NZY177* from the type locality and *BH225* from the flowering population at Shaoguan City of Guangdong Province), two samples of the type species of *Acidosasa*, i.e. *Acidosasaglauca* B. M. Yang (*CZY56* from the type locality of *A.glauca* and *NZY152* from the type locality of *A.chienouensis*, a synonym of *A.glauca*), the type species of *Indosasa*, i.e. *Indosasacrassiflora* McClure and representatives from other genera in Arundinarieae. *Bambusavulgaris* Nees from the tribe Bambuseae was chosen as the outgroup. In total, 31 samples representing 29 species from 19 genera were included with eight newly-sequenced and 23 downloaded from GenBank. For nuclear gene-based phylogenetic inference, a total of 26 samples representing 24 species from 14 genera in the tribe Arundinarieae were included with data of five species unavailable. Voucher information and GenBank accession numbers of plastomes were listed in Suppl. material 1: table S1.

For DNA extractions, young leaves were collected in the field and dried with silica gel. Genomic DNAs were extracted from the dried leaves using the TIANGEN Genomic DNA Extraction Kit (TIANGEN, Beijing, China), following the manufacturer’s instructions and 1 μg DNA per sample was sheared using a Covaris M220 ultrasonicator (Covaris, Woburn, MA). We enriched the resulting 350-bp fragments using PCR and prepared a paired-end library using the NEBNext® UltraTM DNA Library Prep Kit which we sequenced on a NovaSeq 6000 platform. After filtration of adapters and low-quality reads using Fastp software v. 0.23.2 (Chen et al. 2018), at least 40 Gb deep genome skimming (DGS) data were generated.

### ﻿Plastome assembly and nuclear single-copy orthologous genes recovery

We used filtered clean reads to *de novo* assemble complete plastid genomes using the GetOrganelle v. 1.7.6.1 pipeline (Jin et al. 2020), with the plastome of *Phyllostachysedulis* (Carrière) J. Houz. (GenBank accession No. HQ337796) as reference. We set six k-mer values, viz. 21, 45, 65, 85, 105 and 125, for plastid contig assembly. Following assembly, we aligned two generated plastid sequences with opposite short single-copy (SSC) region directions to the reference sequence using Mauve v. 2.4.0 (Darling et al. 2004). We visualised and selected the sequence with the same SSC direction as the reference as the final plastome in the software Geneious v. 9.1.4 (Kearse et al. 2012).

For nuclear genes recovery, we used the protein-coding sequences of six previously published bamboo genomes—*Dendrocalamuslatiflorus* Munro (Zheng et al. 2022), *Phyllostachysedulis* (Carrière) J. Houz. (Zhao et al. 2018), *Boniaamplexicaulis* (L. C. Chia, H. L. Fung & Y. L. Yang) N. H. Xia, *Guaduaangustifolia* Kunth, *Olyralatifolia* L. and *Raddiaguianensis* (Brongn.) Hitchc. (Guo et al. 2019), to identify 737 common nuclear single-copy orthologous genes (SOGs) using Orthofinder v. 2.5.4 (Emms and Kelly 2019). We assembled putative SOGs using HybPiper v. 2.0.1 (Johnson et al. 2016). We mapped filtered clean reads to each SOG using the BWA mapper function in HybPiper. We then *de novo* assembled reads mapped to each gene into contigs with the best k-mer automatically detected by SPAdes v. 3.15.0 (Bankevich et al. 2012). We aligned the assembled contigs to the reference SOG dataset and used a python script ‘retrieve_sequences.py’ to recover 737 putative orthologs for each sample. However, because all of our samples are polyploid (Guo et al. 2019), some so-called SOGs might have multiple copies. We therefore used a python script ‘paralog_retriever.py’ to detect and disregard potential paralogs. After this step, we retained 439 SOGs.

### ﻿Alignment construction and phylogenetic inference

We aligned the plastid genomes and 439 SOGs using MAFFT v. 7.505 (Katoh and Standley 2013) in the software Geneious. We trimmed each single-gene matrix using trimAl v.1.4 (Salvador et al. 2009) with default settings. We then removed those nuclear genes with lengths shorter than 300 bp or with > 25% missing data. The final nuclear dataset used for phylogenetic analyses included 433 conserved nuclear genes.

As the plastome is a linkage group without recombination (Doyle 2022), we thus performed Maximum Likelihood (ML) analysis for plastid DNA data. We ran the multispecies coalescent-based method for phylogenetic inference for the nuclear dataset as different nuclear genes possess heterogeneous nucleotide substitution rates (Doyle 1992; Maddison 1997).

For plastome-based phylogenetic inference, we used RAxML v. 8.2.12 (Stamatakis 2014) to perform 20 addition replicates under the GTR+Γ model. We chose the GTR+Γ model because it accommodates rate heterogeneity amongst sites, while the other available GTR models in RAxML are less appropriate due to the small taxon sampling size (Stamatakis 2014; Cai et al. 2021). We estimated branch support using a rapid bootstrap algorithm with 1000 bootstrap replicates. For nuclear gene-based phylogenetic inference, we inferred individual ML trees using RAxML for each nuclear gene and estimated branch support using bootstrapping analysis with 500 replicates, all using the GTR+Γ model. We tested different thresholds by collapsing branches with support < 30% and 50% and compared the resulting trees to the tree without collapsed branches. This procedure was applied to each ML bifurcation locus tree by Newick Utilities (Junier and Zdobnov 2010). We combined all the generated bifurcation trees to infer the species tree using ASTRAL-III (Zhang et al. 2018). The local posterior probability was calculated with the parameter ‘-t 3’.

## ﻿Results

### ﻿Morphological study

**Figure 1.**
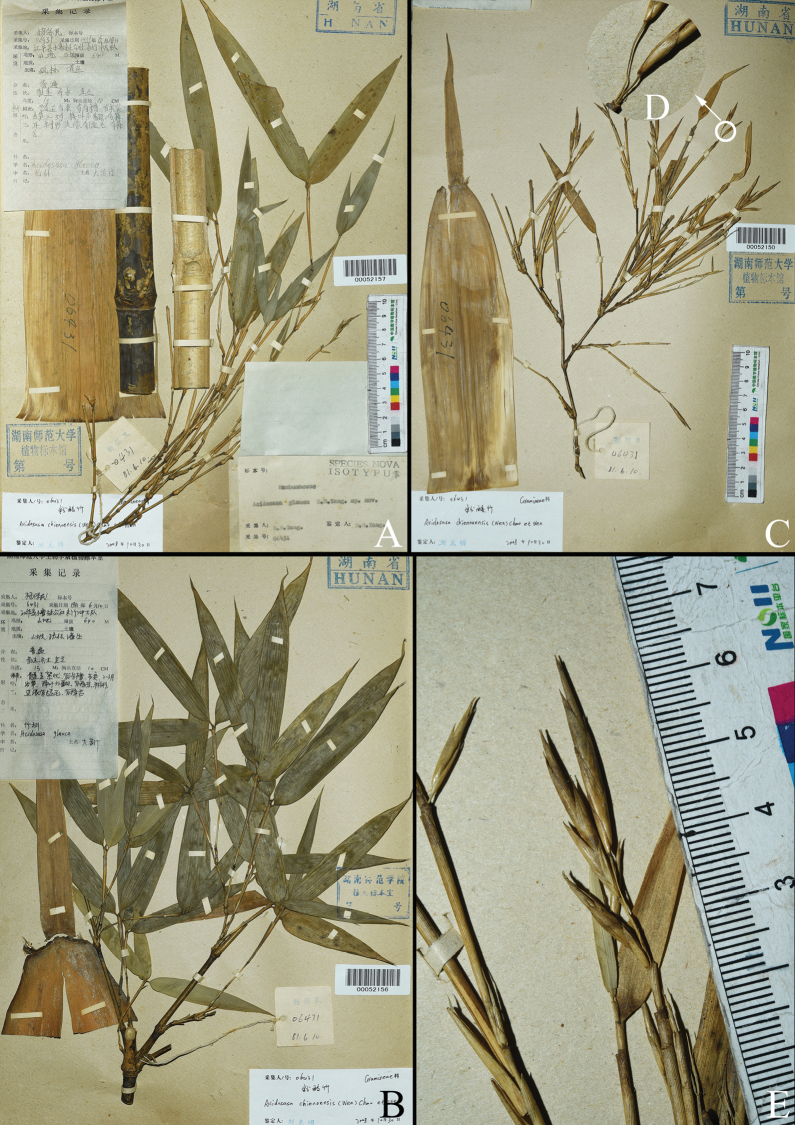
Isotypes of *A.glauca* B. M. Yang (*B. M. Yang 06431*, HNNU) **A, B** sheets with vegetative part **C** sheet with flowering branches and a culm leaf **D** spikelet pedicels **E** flowering branch.

**Figure 2.**
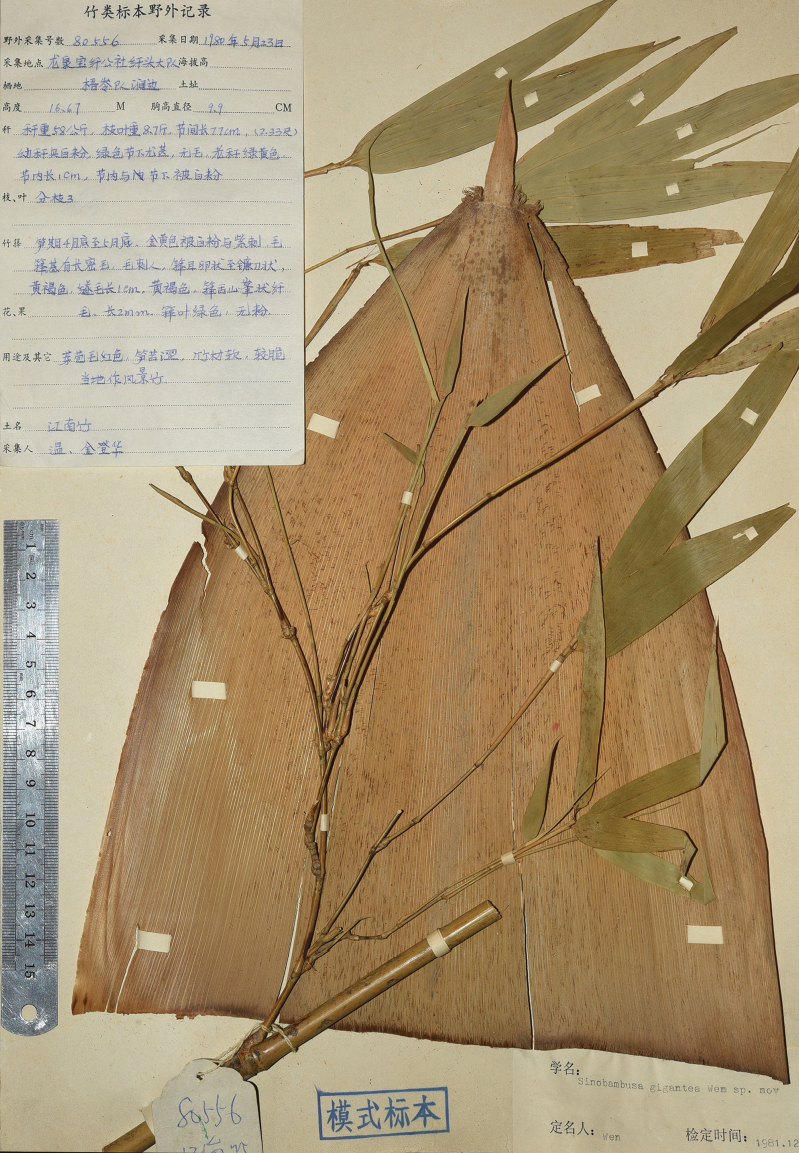
Holotype of *Indosasagigantea* (T. H. Wen) T. H. Wen (*T. H. Wen & D. H. Jin Wen80556*, ZJFI).

**Figure 3.**
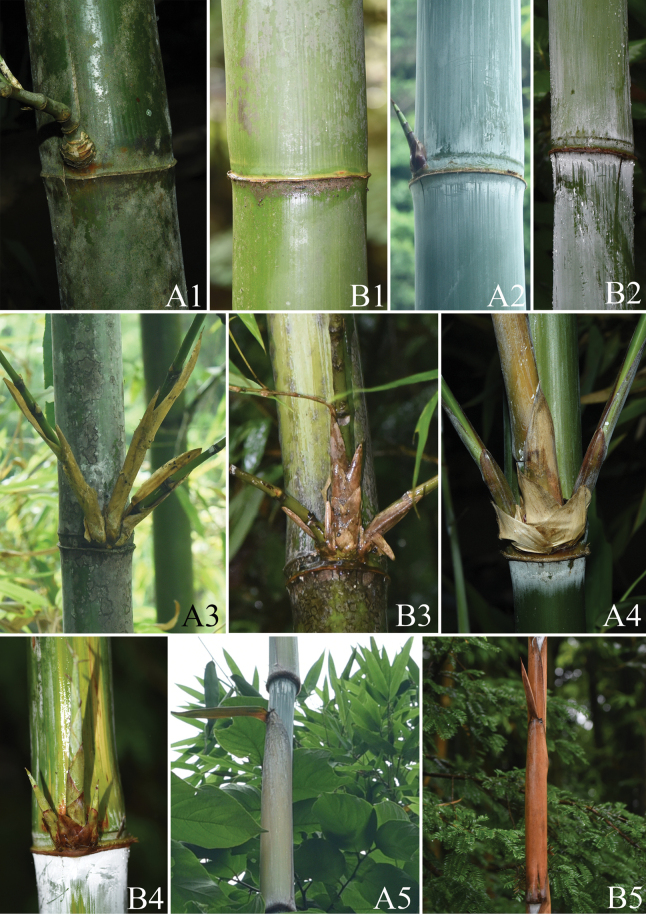
Morphological comparison of *Acidosasaglauca* (**A**) and *Indosasagigantea* (**B**). **A1**–**B1** old culms **A2**–**B2** young culms **A3**–**B3** three branches at old mid-culm node **A4**–**B4** three branches at young mid-culm node **A5**–**B5** culm leaves.

**Figure 4.**
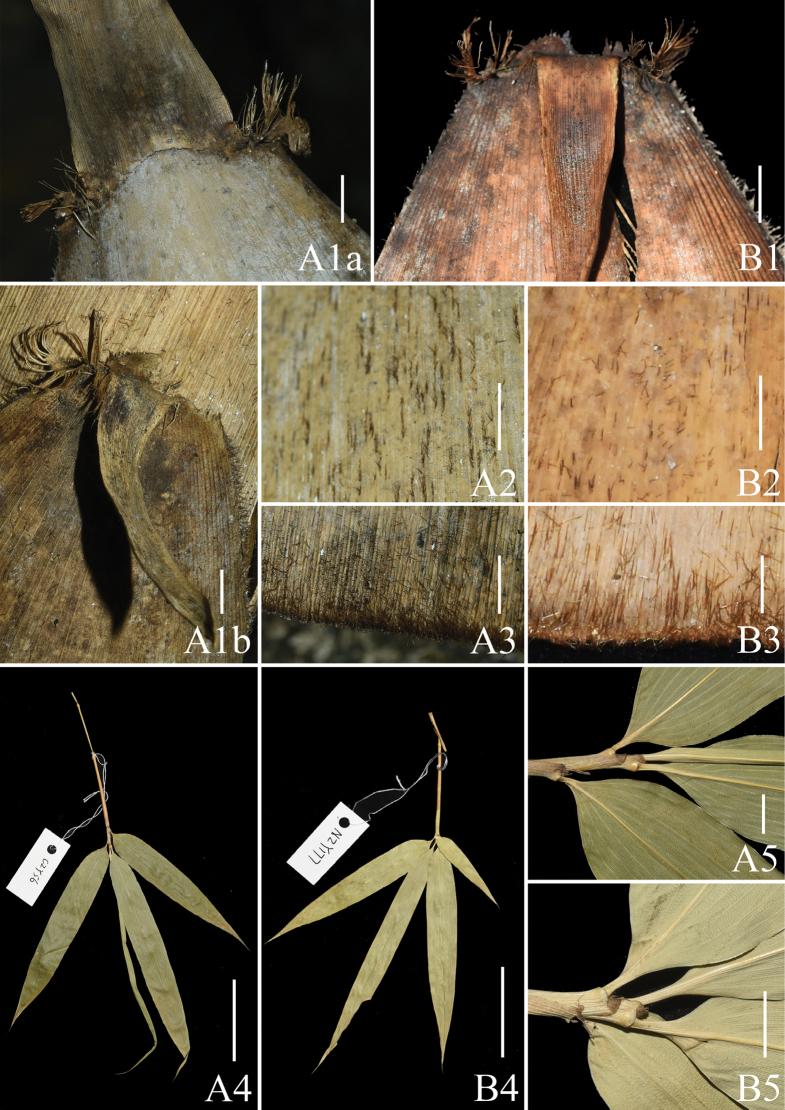
Morphological comparison of *Acidosasaglauca* (**A**) and *Indosasagigantea* (**B**). **A1**–**B1** culm leaf apex, showing auricles, oral setae, ligules and blades **A2**–**B2** abaxial surface of culm leaf sheaths covered with sparse brown setae and white powder **A3**–**B3** bases of abaxial surface of culm leaf sheaths covered with dense brown setae **A4**–**B4** ultimate foliage leafy branches **A5**–**B5** foliage leaf sheath and ligules. Scale bars: 1 cm (**A1**–**A3**, **B1**–**B3**); 5 cm (**A4**–**A5**); 5 mm (**A5**–**B5**).

A detailed morphological comparison between *Indosasagigantea* and *Acidosasaglauca* was conducted, based on examination of type specimens, critical analysis of descriptions in the protologues and observations in the field. The results showed that the two species share exactly the same key characters, such as the thickly powdery young culms, branch complement with three branches at each mid-culm node, sparsely brown setose and white-powdery abaxial surface of culm leaf sheaths with a densely brown setose base, ovate to falcate culm leaf auricles with many radiating or sometimes curly oral setae, prominent culm leaf ligules, narrow triangular to lanceolate culm leaf blades, 3 or 4 foliage leaves per ultimate branch, glabrous foliage leaf sheaths, undeveloped foliage leaf auricles usually without oral setae or with several oral setae at the most basal leaf sheath apex and foliage leaf blades being glabrous adaxially and pubescent abaxially (Table 1, Figs 1–4). The only difference seems to be the colour of culm leaf sheaths: *I.gigantea* has pale red-brown abaxial surface of culm leaf sheaths (Fig. 3B5), while that of *A.glauca* is green to yellow-brown (Fig. 3A5). However, the colour of culm leaf sheaths seems to be unstable, which is easily affected by the light condition of the habitats according to our observations in the wild. Specifically, with a strong light condition, the culm leaf sheaths will often be redder than those within weak light conditions.

**Table 1.** Comparison of key morphological characters between *Indosasagigantea* and *Acidosasaglauca*.

Characters	*I.gigantea*	*A.glauca*
Young culm	Glabrous, with thick white powder	Glabrous, with thick white powder
Number of branches at mid-culm node	three	three
**Culm leaf**		
Colour of sheath	Pale red-brown	Yellow-brown
Abaxial surface of sheath	Sparsely brown setose and white powdery, densely brown setose at base	Sparsely brown setose and white powdery, densely brown setose at base
Auricles	Ovate to falcate	Falcate
Oral setae	Radiating, ca. 1 cm long	Radiating or curly, ca. 0.8 cm long
Ligule	3–5 mm high, prominent	2–3 mm high, prominent
Shape of blade	Narrow triangular to lanceolate	Narrow triangular to lanceolate
Number of foliage leaves per ultimate branch	3 or 4	3 or 4
**Foliage leaf**		
Abaxial surface of sheath	Glabrous	Glabrous
Auricles	Absent	Absent
Oral setae	Usually absent or several at the most basal leaf sheath apex	Usually absent or several at the most basal leaf sheath apex
Abaxial surface of blade	Pubescent	Pubescent
Adaxial surface of blade	Glabrous	Glabrous

**Figure 5.**
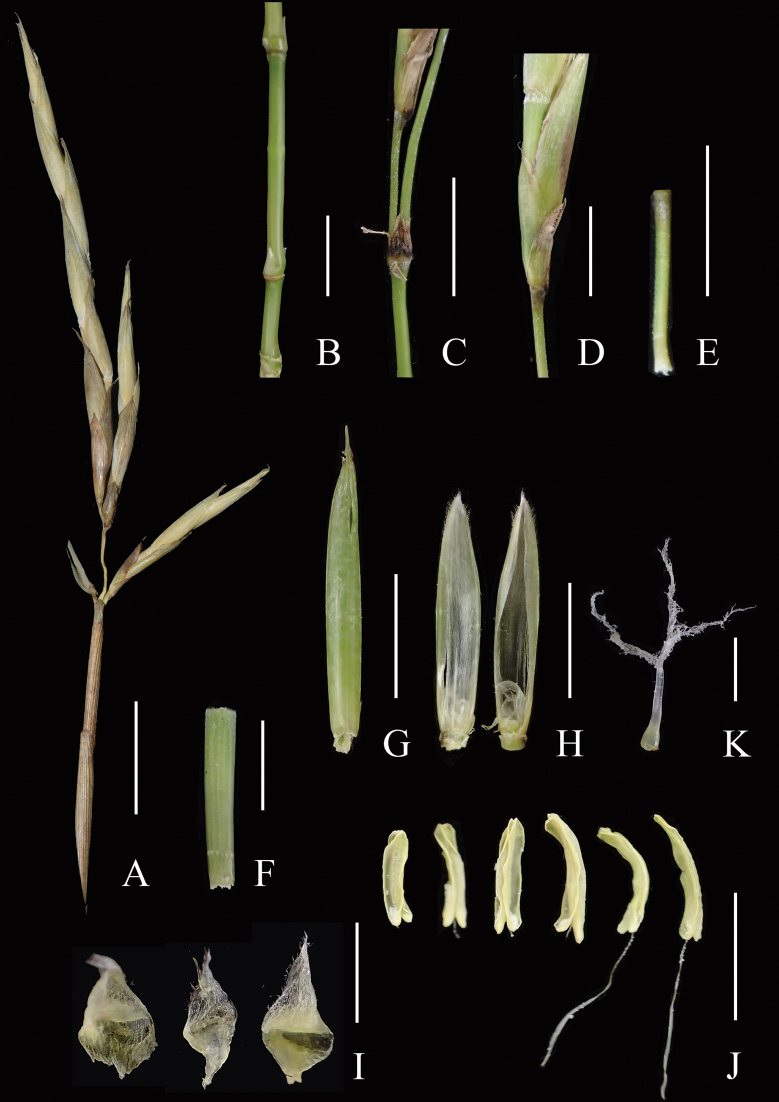
Dissection of inflorescence of *Indosasagigantea* (voucher: *BH225*, IBSC) **A** flowering branch **B** internodes of lower part of flowering branch **C** small membranous bract at the base of spikelet pedicel **D** base of spikelet, showing two glumes and a floret **E** spikelet pedicel **F** rachilla segment **G** lemma, abaxial view **H** palea, adaxial (left) and abaxial (right) view **I** lodicules **J** stamens, with two possessing filaments **K** pistil. Scale bars: 1 cm (**A**); 5 mm (**B–H**, **J**); 2 mm (**K–I**).

The flowering materials found at Shaoguan City of Guangdong Province have raceme-like inflorescence with (1–)2–5 pedicellate spikelets, two glumes, each spikelet with several to over ten florets, pubescent rachilla segments, glabrous and 11–13-veined lemma, palea with the ciliate upper parts of keels and acute apex, three lodicules, six stamens with 4–5 mm long anthers and ovary with one style and three stigmas (Fig. 5).

### ﻿Phylogenetic study

**Figure 6.**
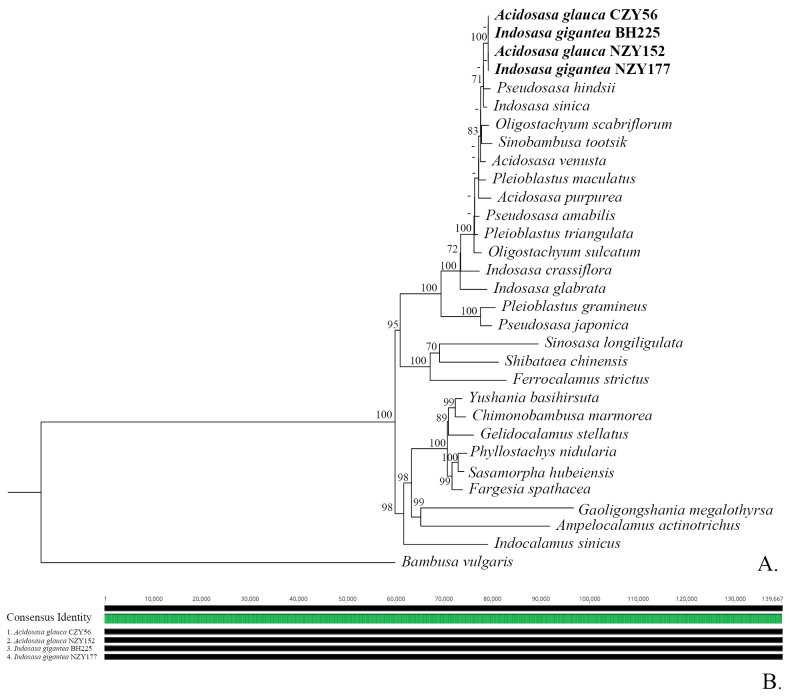
**A** The phylogram of 28 species belonging to 18 genera from Arundinarieae, based on plastome sequences. The bootstrap values ≥ 70% are shown around the branches, while those values < 70% are represented by the hyphens **B** the plastid genome sequence alignment of two samples of *A.glauca* and two samples of *I.gigantea*, showing that the four plastomes are totally identical.

The basic features of the plastomes of all the samples in our study are summarised in Suppl. material 1: table S2. The plastid genome sequence alignment, based on two samples of *Acidosasaglauca* and two samples of *Indosasagigantea*, is green in all sites, which means that the four plastid genomes are identical (Fig. 6B). The size of the plastome is 139,677 bp, including a large single-copy (LSC) region with 83,261 bp, a short single-copy (SSC) region with 12,816 bp and one pair of inverted repeats with 21,795 bp (Suppl. material 1: table S2).

**Figure 7.**
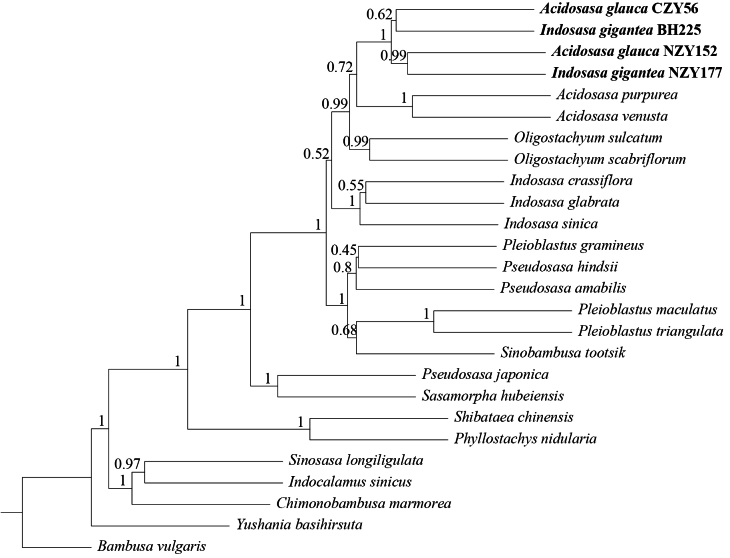
The ASTRAL species tree of 23 species belonging to 15 genera of the tribe Arundinarieae, which is reconciled by coalescence of 433 single-copy orthologous nuclear gene trees after collapsing branches with support < 30%. The posterior probabilities are shown around the branches.

Both *Acidosasa* and *Indosasa* are resolved as polyphyletic in the plastome-based tree (Fig. 6) and nuclear SOG-based species tree (Fig. 7). In the plastome-based tree, the two samples of *I.gigantea* and the two samples of *A.glauca* are clustered into a monophyletic clade with high support values (BS = 100) and without any branch length in their interiors (BS < 70). The nuclear SOG-based species tree also strongly supported (PP = 1.0) that *I.gigantea* is clustered with *A.glauca*, the type species of *Acidosasa*, but distantly related to *I.crassiflora*, the type species of *Indosasa*.

## ﻿Discussion

The morphological characters of the specimens collected in the field (vouchers: *CZY56* and *NZY177*) matched well with the description of *Indosasagigantea* made by Wen (1991) (except the type of spikelet) and the holotype of *Acidosasaglauca* (Table 1, Figs 1–4). Our phylogenetic study also suggested that *I.gigantea* is a member of *Acidosasa* rather than *Indosasa*. Wen (1991) did describe “indeterminate inflorescence” for *Indosasagigantea*, which is a key character of *Indosasa*, but its flowering materials found at IBSC (voucher: *BH225*) are diagnosed by the possession of spikelet pedicels. Thus, we argue that the transfer of this species from *Indosasa* to *Acidosasa* by Xie and Chen is correct, although we did not find the flowering specimen mentioned by them.

Although there are multiple instances of conﬂict between the plastid and nuclear SOG-based trees, the plastid and nuclear sequences both strongly support that two samples *Acidosasaglauca* and two samples of *Indosasagigantea* intermingle together (Figs 6, 7). We also noted that *A.chienouensis*, which is the synonym of *A.glauca*, is always clustered with *I.gigantea*, whether based on several plastid molecular markers (Zeng et al. 2010) or single nuclear gene (Zhang et al. 2012). Hence, our phylogenetic studies are congruent with previous studies on the relationships between *A.glauca* and *I.gigantea*.

Our morphological and phylogenetic evidence further supported that *Indosasagigantea* is conspecific with *Acidosasaglauca* (Figs 1–7, Table 1). As *Acidosasaglauca* B. M. Yang (1981) predates the basionym of *I.gigantea*, i.e. *Sinobambusagigantea* T. H. Wen (1983), the latter is thus proposed as a synonym of *A.glauca* here according to Art. 11.4 of International Code of Nomenclature for algae, fungi and plants (Shenzhen Code) (Turland et al. 2018).

### ﻿Taxonomic treatment

#### ﻿ Acidosasa glauca

Taxon classificationPlantaePoalesPoaceae

B. M. Yang, J. Hunan Teachers’ Coll. (Nat. Sci. Ed.) 1981(2): 54 (1981)

8B6EA945-8FA2-5114-9789-D7BB2489DC11

= Indosasagigantea (T. H. Wen) T. H. Wen, J. Bamboo Res. 10(1): 22 (1991). syn. nov. ≡ Sinobambusagigantea T. H. Wen, J. Bamboo Res. 2(1): 57, fig. 10 (1983). ≡ Acidosasagigantea (T. H. Wen) Q. Z. Xie & W. Y. Zhang, Bull. Bot. Res., Harbin 13(1): 74 (1993). Type: CHINA • Zhejiang: Longquan City, Baoxi Town, Wuling Village, 23 May 1980, *T. H. Wen & D. H. Jin Wen80556* (holotype: ZJFI!).
= Acidosasachienouensis (T. H. Wen) C. S. Chao & T. H. Wen, J. Bamboo Res. 7(1): 31 (1988). ≡ Indosasachienouensis T. H. Wen, J. Bamboo Res. 2(1): 67, fig. 19 (1983). Type: CHINA • Fujian: Chien’ou [Jian’ou] County, Wanmulin, 2 June 1981, *X. Q. Hua & P. X. Zhang FJ81607* (holotype: ZJFI!).

##### Type.

China • Hunan: Jianghua County, Weizhuchong Town, Weizhuchong Village, 10 June 1981, *B. M. Yang 06431* (holotype: HNNU; isotypes: HNNU!).

##### Description.

Running bamboo. Rhizomes leptomorph. Culms diffuse, erect, 7–17 m tall and 4–10 cm in diameter; internodes terete, 30–70 cm long, green, thickly white powdery when young, glabrous; supra-nodal ridges weakly prominent or flattened; sheath scars prominent, with a ring of dense brown setae when young, glabrescent when old. Mid-culm branch complement with three branches. Culm leaf sheaths initially pale green, turning to gold-yellow, yellow-brown or pale red-brown when old, caducous, triangular, thickly leathery, abaxially sparsely brown setose and white powdery, base densely brown setose, margins densely ciliate; auricles ovate to falcate, 3.5–10 × 2–6 mm; oral setae well-developed, many, scabrid, radiating or curly, 0.5–1 cm long; ligules fragile, arcuate, 2–4 mm tall, abaxially pubescent, apex with deciduous short cilia; blades erect or reflexed, easily deciduous, narrowly triangular to lanceolate, 2–12.5 × 0.7–3 cm, apex acuminate, base broadened or slightly narrowed. Foliage leaves 3 or 4 per ultimate branch; sheaths 4–6.5 cm long, glabrous, longitudinal ribs conspicuous; auricles absent; oral setae usually absent or several at the lowest one or two sheath apex; ligules truncate, 1–2 mm tall, abaxially pubescent; blades lanceolate, papery, 5–15 × 0.8–2 cm, base subrounded or cuneate, abaxially pubescent, adaxially glabrous, both margins serrulate, secondary veins 4–6 pairs, transverse veins conspicuous, margins serrulate. Inflorescence raceme-like, with (1–)2–4 spikelets, axis glabrous, without white powder, basal internodes 6–8 mm long, glabrous; spikelet pedicels 3–12 mm long, glabrous, without white powder, basally subtended by a small membranous bract. Spikelets slightly laterally compressed, (1.5–)5–7.5 cm long, fertile florets 3–12(–15), uppermost one not fully developed; rachilla segments compressed, 5–10 mm long, pubescent, with several longitudinal ridges; glumes 2, first glume narrowly triangular, ca. 8 mm long, apically pubescent or glabrous, 1–3-veined, apex acute; second glume ovate to lanceolate, ca. 11 mm long, indumentum the same as the first glume, 7–9-veined; lemma lanceolate, ca. 13 mm long, abaxially sparsely pubescent at the upper parts, while glabrous at other parts or glabrous wholly, white powdery, 11–15-veined, apex acuminate; palea shorter than or equal to lemma, 9–13 mm long, 2-keeled, keels white ciliate on the upper parts, 4 or 5-veined between keels, 3-veined outside keels each side, apex acute; lodicules 3, 2.5–4 × 1–1.4 mm, ovate, the upper parts membranous, while middle and lower parts fleshy, margins sparsely ciliate; stamens 6, anthers initially yellow, brown when old, 4–5 mm long, filaments ca. 4 mm long; ovary ovate, ca. 1 mm long; style 1, 2–2.5 mm long; stigmas 3, plumose. Caryopsis unknown.

##### Distribution and habitat.

Up to now, this species has been known native to north Fujian and widely cultivated in Zhejiang, Guangdong and Hunan. It prefers sunny environments and often grows well on mountain slopes near roadsides, creeks and farmlands.

##### Phenology.

New shoots March to April. Flowers April to June.

##### Chinese names.

Chinese name 粉酸竹 [fěn suān zhú]; “橄榄竹” [gǎn lǎn zhú].

##### Additional specimens examined.

**China • Fujian**: Chien’ou City, Jiubao Village, Jiangdangping, 1 June 1981, *P. X. Zhang & X. Q. Hua FJ81606* (ZJFI); • Chien’ou City, the nursery of Wanmulin, 27°2'54"N, 118°8'33"E, 2 June 2022, *Z. Y. Niu NZY152* (IBSC); • Jiangle County, Longqishan, 24 May 1981, *P. X. Zhang & X. Q. Hua FJ81536* (ZJFI). **Guangdong**: • Fengkai County, Heishiding, 12 April 1982, *M. Y. Xiao 31853* (CANT); • Shaoguan City, Zhangshi Town, Luxi Village, 24°28'55"N, 113°27'27"E, 16 April 2023, *Y. H. Tong, J. B. Ni & D. H. Cui BH225* (IBSC); Shixing County, Chebaling, 18 April 2023, 24°46'3"N, 114°18'3"E, *Y. H. Tong, J. B. Ni & D. H. Cui BH238* (IBSC); • Yunan County, Gaoliang Service Area, 13 April 2023, 23°14'30"N, 111°54'16"E, *Y. H. Tong, J. B. Ni & D. H. Cui BH209* (IBSC). **Hunan**: • Jianghua County, Weizhuchong Town, roadside near Xiaoluguikou County Road 085, 24°53'10"N, 111°48'37"E, 19 May 2019, *Z.Y. Cai CZY56* (IBSC). **Zhejiang**: • Longquan City, Baoxi Town, Wuling Village, 28°0'27"N, 118°46'29"E, 20 June 2022, *Z. Y. Niu NZY177* (IBSC).

##### Local usage.

Its culms can be used for construction. Its shoots are edible and tasty. It is very suitable for landscape due to the elegant architecture and appearance.

## Supplementary Material

XML Treatment for Acidosasa glauca

## ﻿Additional information

### Author ORCIDs

Zhengyang Niu https://orcid.org/0000-0003-0281-1504

Zhuoyu Cai https://orcid.org/0000-0001-9288-0882

Yihua Tong https://orcid.org/0000-0002-5034-005X

Nianhe Xia https://orcid.org/0000-0001-9852-7393

### Data availability

All of the data that support the findings of this study are available in the main text or Supplementary Information.

## Supplementary materials

Supplementary material 1 433 single_copy orthologous gene alignments used for the reconstruction of ASTRAL species tree and two supplementary tables Data type zip Explanation note Here are 433 single_copy orthologous gene alignments used for the reconstruction of ASTRAL species tree, the basic features of a total of 31 plastomes and plastome and nuclear datasets and its corresponding specimen voucher information used in our study. https://binary.pensoft.net/file/1314266This dataset is made available under the Open Database License (http://opendatacommons.org/licenses/odbl/1.0/). The Open Database License (ODbL) is a license agreement intended to allow users to freely share, modify, and use this Dataset while maintaining this same freedom for others, provided that the original source and author(s) are credited.Zhengyang Niu
